# An Acidic Exopolysaccharide from *Haloarcula hispanica* ATCC33960 and Two Genes Responsible for Its Synthesis

**DOI:** 10.1155/2017/5842958

**Published:** 2017-05-28

**Authors:** Yang Lü, Hua Lu, Shiwei Wang, Jing Han, Hua Xiang, Cheng Jin

**Affiliations:** ^1^State Key Laboratory of Mycology, Institute of Microbiology, Chinese Academy of Sciences, Beijing, China; ^2^University of Chinese Academy of Sciences, Beijing, China; ^3^State Key Laboratory of Microbial Resources, Institute of Microbiology, Chinese Academy of Sciences, Beijing, China; ^4^Guangxi Academy of Sciences, Nanning, Guangxi, China

## Abstract

A 1.1 × 10^6^ Da acidic exopolysaccharide (EPS) was purified from an extremely halophilic archaeon *Haloarcula hispanica* ATCC33960 with a production of 30 mg L^−1^ when grown in AS-168 medium, which mainly composed of mannose and galactose with a small amount of glucose in a molar ratio of 55.9 : 43.2 : 0.9. Two glycosyltransferase genes (*HAH_1662* and *HAH_1667*) were identified to be responsible for synthesis of the acidic EPS. Deletion of either *HAH_1662* or *HAH_1667* led to loss of the acidic EPS. The mutants displayed a different cell surface morphology, retarded growth in low salty environment, an increased adhesion, and swimming ability. Our results suggest that biosynthesis of the acidic EPS might act as an adaptable mechanism to protect the cells against harsh environments.

## 1. Introduction

Microorganisms produce EPSs as a strategy for growing, adhering to solid surfaces, protective barrier, a reserve nutrient, and formation of a biofilm as an adaptive lifestyle to encourage the survival in harsh environments and under changing environmental conditions [1–4]. EPS produced by bacteria has a wide range of potential applications in many industrial fields in which emulsifying, viscosifying, antioxidant, and chelating agents are required [5–7].

In order to find EPSs with novel and valuable properties, several EPSs from haloarchaea have been isolated and investigated, such as *Haloferax*, *Halococcus*, *Haloarcula*, *Natronococcus*, *Haloterrigena*, and *Halobacterium* [8–12]. The structures of several haloarchaeal EPSs have been solved but little is known about their biosynthesis [13]. The repeat unit of EPS from *Haloferax gibbonsii* ATCC33959 contains one main chain and two branches. The main chain is composed of two mannosyl and two galactosyl moieties; one branch contains one glucosyl moiety and the other branch is composed of one galactosyl and one rhamnosyl moiety [10]. The EPS from *Haloferax mediterranei* ATCC 33500 was identified to be a heteropolysaccharide containing mannose as the major component [14]. The repeat unit of EPS in *H. mediterranei* contains one mannosyl and two N-acetyl-glucosaminuronyl moieties, and one N-acetyl-glucosaminuronyl group is modified by a sulfonic group [15]. Based on the complete genome sequence of *H. mediterranei*, a gene cluster involved in EPS biosynthesis in *H. mediterranei* was identified [16]. Deletion of the gene cluster eliminated EPS synthesis. The mutant strain deficient of EPS biosynthesis showed a remarkable decrease in viscosity and foaming propensity of culture broth, increase in content of dissolved oxygen, and enhanced production of PHBV [17].

*Haloarcula hispanica* is an extremely halophilic archaeon, originally isolated from a solar saltern in Spain, and a producer of an extracellular polymer that gave a typical mucous character of the colonies [18]. *Har. hispanica* displays particularly low restriction activity and is therefore one of the most tractable haloarchaea for archaeal genetic research [19]. In this study, we isolated and purified an acidic EPS from *Har. hispanica* ATCC33960. By the gene deletion method, *HAH_1662* and *HAH_1667* were identified to be responsible for biosynthesis of this acidic EPS. Also, the impact of the acidic EPS on growth of *Har. hispanica* was evaluated.

## 2. Materials and Methods

### 2.1. Strains and Culture Conditions

*Haloarcula hispanica* ATCC 33960 and its mutant strains were cultured in AS-168 medium (per 1 L, 5 g Bacto Casamino acids, 5 g Bacto yeast extract, 1 g sodium glutamate, 3 g trisodium citrate, 20 g MgSO_4_·7H_2_O, 2 g KCl, 200 g NaCl, 50 mg FeSO_4_·7H_2_O, 0.36 mg MnCl_2_·4H_2_O, pH 7.0). Plates contained 1% agar unless mentioned otherwise. Mevinolin (Sigma) was added to a final concentration of 5 *μ*g/mL in AS-168 medium for the screening of pUBP pop-in strains and pWL102 complementary strains. For transformation of pWL-CBD plasmid, novobiocin (Calbiochem) was added to a final concentration of 0.16 *μ*g/mL in AS-168 medium. *Escherichia coli* JM110 was grown in LB medium. When needed, ampicillin was added to a final concentration of 100 *μ*g/mL for *E. coli*.

### 2.2. Isolation and Purification of EPS

The cells were cultured in 1 L AS-168 medium to late stationary phase and removed from the culture broth by centrifugation at 13000 ×g for 30 min at 4°C. The EPS was precipitated from the supernatant by the addition of fourfold volume of cold ethanol at 4°C overnight. The precipitation was collected by centrifugation at 13000 ×g for 30 min and resolved in water. Then, the solution was dialyzed against water (molecular weight cutoff 14 kDa) to get rid of salts for 2 days, by which most halophilic proteins were denatured and precipitated, the dialyzed solution was centrifugated at 13000 ×g for 30 min and the supernatant was treated with 10 *μ*L Benzonase nuclease (Sigma, ≥250 units/*μ*L, MW 30 kDa) at 37°C for 12 h, prior to treatment with 3 mg protease K (Sigma, ≥30 units/mg, MW 29 kDa) at 37°C for 12 h, and then, the supernatant was concentrated fivefold with 100 kDa ultrafiltration membrane (Millipore) and lyophilized.

The crude EPS was solubilized at 10 mg/mL with buffer A (20 mM sodium acetate) and passed through an anion exchange column DEAE-Sepharose Fast Flow (Sigma). After washing with buffer A, EPS bound to the column was eluted by a linear gradient ranging from buffer A to buffer B (20 mM sodium acetate, 2.5 M sodium chloride) in 5 column volumes at a flow rate of 0.5 mL/min. The EPS emerged from the column in fractions from 1.25 to 1.55 M NaCl. The elution fractions were monitored by UV detection at 280 nm, the phenol-sulfuric acid reaction [20], and acidic polysaccharide electrophoresis. The acidic polysaccharides were separated in 7.5% PAGE gel and dyed with 0.5% methylene blue in 3% acetic acid.

Then, the acidic EPS fractions were pooled, dialyzed against water, and lyophilized. The lyophilized EPS was solubilized with water at 2 mg/mL and loaded onto Sephacryl S-400/HR (GE Healthcare) column and eluted with water at a flow rate of 0.5 mL/min. Fractions were monitored by phenol-sulfuric acid reaction. The main peak fractions were pooled and lyophilized; the yield of the acidic EPS was determined by weight.

### 2.3. Homogenity and Molecular Weight

The homogeneity and molecular weight of polysaccharide were estimated by HPGPC with TSK G4000 column and refractive index detector, eluted with mobile phase containing 0.1 M NaNO_3_ at a flow rate of 0.5 mL/min. The column temperature was kept at 30°C. For molecular weight estimation, the column was calibrated by standard dextrans (50 kDa, 80 kDa, 150 kDa, 270 kDa, 410 kDa, 670 kDa, and 1100 kDa). All samples were prepared as 3 mg/mL solution, and 40 *μ*L of solution was analyzed in each run.

### 2.4. Glycosyl Composition Analysis

The composition analysis was performed on 200 *μ*g of pure EPS. As internal standard, 20 *μ*g inositol was added to the samples. The sample was hydrolyzed in 2 M trifluoroacetic acid (TFA) for 2 h in a sealed tube at 121°C, reduced with NaBD_4_, and acetylated using acetic anhydride/TFA. The resulting alditol acetates were analyzed on Agilent 7890A GC interfaced to a 5975C MSD, electron impact ionization mode. Separation was performed on a 30 m Supelco SP-2331 bonded phase fused silica capillary column.

### 2.5. Sulfate Content Analysis

2 mg EPS was hydrolyzed in 2 M TFA for 2 h in a sealed tube at 121°C. The hydrolysis products were vacuumly dried and then solubilized with H_2_O. The presence of sulfate was attested by using a high-performance liquid ion chromatography system (ICS-2100, Thermo Scientific) equipped with an IonPac AS11-HC column. A solution of 30 mM NaOH was used as eluent at a flow rate of 1 mL/min. The column temperature was kept at 30°C. A calibration curve prepared with Na_2_SO_4_ as a standard was used to calculate the sulfate content in the EPS.

### 2.6. Construction and Confirmation of the Deletion Mutant and Reverted Strains

Chromosomal deletions were generated by using a homologous recombination (pop-in/pop-out) method as previously described [21]. The sequence of PCR primers used in this study was summarized in Table 1. A 606 bp upstream and a 618 bp downstream flanking sequences of the *HAH_1662* were amplified by PCR using primer pairs of p1/p2 and p3/p4. Two amplified DNA fragments were linked using overlap PCR with primer pairs of p1 and p4 to generate a 1.2 kb fragment containing a *Hind* III site at 5′ end and a *Kpn* I site at 3′ end. The fragment was then cloned into the pUBP plasmid between the *Hind* III and *Kpn* I sites to yield the pUBPΔHAH_1662 plasmid. The plasmid was then transformed into *Har. hispanica* wild-type cells and plated onto AS-168 solid medium containing mevinolin. Transformants were screened for integration of the gene knockout plasmid at the corresponding locus by PCR analysis. Cells with pUBPΔHAH_1662 integrated into their genome were subcultured at least three times in AS-168 medium without mevinolin to allow an occurrence of the second recombination. For reverse complementation, the plasmid pWL-CBD-SecY (a gift from Professor Jerry Eichler) was digested with *Nde* I and *Kpn* I to remove the *SecY* gene [22]. The generated pWL-CBD fragment contained a constitutive promoter PrR16 and the cellulose binding domain from *Clostridium thermocellum*. The *HAH_1662* gene was amplified using primer pairs p5 and p6 (Table 1), in which *Nde* I and *Kpn* I restriction sites were introduced at the start and end of the gene, respectively. The amplified fragment was cloned into pWL-CBD at the *Nde* I-*Kpn* I site to generate the CBD fusion expression plasmid pWL-CBD-HAH_1662. Confirmation of the Δ*HAH_1662* was carried out by digestion of the genomic DNA with *EcoR* V. The 1550 bp downstream fragment of the *HAH_1662* gene amplified by primers p7 and p8 was used as a probe.

A 594 bp upstream and a 613 bp downstream flanking sequences of the *HAH_1667* were amplified by PCR using primer pairs of p9/p10 and p11/p12. Two amplified DNA fragments were linked using overlap PCR with primer pairs of p9 and p12 to generate a 1.2 kb fragment containing a *Hind* III site at 5′ end and a *Kpn* I site at 3′ end. The fragment was then cloned into the pUBP plasmid between the *Hind* III and *Kpn* I sites to yield the pUBPΔHAH_1667 plasmid. The plasmid was then transformed into *Har. hispanica* wild-type cells and plated onto AS-168 solid medium containing mevinolin. Transformants were screened for integration of the gene knockout plasmid at the corresponding locus by PCR analysis. Cells with pUBPΔHAH_1667 integrated into their genome were subcultured at least three times in AS-168 medium without mevinolin to allow an occurrence of the second recombination. For reverse complementation, the plasmid pWL102 was digested with *BamH* I and *Kpn* I. The *HAH_1667* gene with the upstream 200 bp and downstream 100 bp was amplified using primer pairs p13 and p14, in which *BamH* I and *Kpn* I restriction sites were introduced. The amplified fragment was cloned into pWL102 at the *BamH* I-*Kpn* I site to generate the expression plasmid pWL102-HAH_1667. Generated plasmid was then transformed into the *HAH_1667* deletion cells. Confirmation of the Δ*HAH_1667* was carried out by digestion of the genomic DNA with *EcoR* I. The 1408 bp downstream fragment of the *HAH_1667* gene amplified by primers p15 and p16 was used as a probe. Labeling and visualization were performed using the DIG DNA labeling and detection kit (Roche Applied Science) according to the manufacturer's instruction.

### 2.7. RT-PCR

Strains were grown in AS-168 medium at 37°C with shaking at 200 rpm. When OD_600 nm_ reached 0.6–0.8, cells were harvested and total RNA was extracted using Trizol (Invitrogen). Contaminating DNA was removed with RNase-free DNase (New England Biolabs). cDNA was synthesized from the corresponding RNA using random hexamers in a RevertAid First Strand cDNA Synthesis Kit (Thermo Scientific). The single-strand cDNA was then used as templates in PCR reaction containing the appropriate sense and antisense primers for each gene. The sequences of PCR primers used in RT-PCR were summarized in Table 1. Primers p17 and p18 are designed for *HAH_1662*, primers p19 and p20 are designed for *HAH_1667*, primers p21 and p22 are designed for cellulose-binding domain of the expression plasmid pWL-CBD-HAH_1662, and primers p23 and p24 are designed for the mevinolin resistance gene of the expression plasmid pWL102-HAH_1667. These RT-PCR products were separated in 1% agarose gel, followed by ethidium bromide staining.

### 2.8. Scanning Electron Microscopy (SEM) Analysis

Strains were cultured on AS-168 solid plate medium at 37°C for 5 days and then scraped off with a sterilized toothpick into 500 *μ*L 21% salt solution containing 3% glutaraldehyde, mixed uniformly and stand overnight at 4°C. Cells were washed 3 times in 21% salt solution, dehydrated for 15 min in 30%, 50%, 70%, 85%, 95%, and 100% ethanol sequentially, and CO_2_ critical point dried. The lyophilized strains were fixed to the SEM stubs with double side carbon tape and then coated with a layer of platinum. The sample was observed in a cold field emission scanning electron microscope (SU8010, Hitachi Ltd., Japan).

### 2.9. Negatively Stained Transmission Electron Microscopy (TEM) Analysis

Strains were cultured on AS-168 solid plate medium at 37°C for 5 days and then scraped off with a sterilized toothpick into 500 *μ*L 21% salt solution and mixed uniformly. Put the carbon-coated grids in a petri-dish with carbon side facing up and put the petri-dish in a glow-discharge to make the grid surface hydrophilic. Then, 10 *μ*L of sample solution was put on the glow-discharged surface of the carbon grid for about 1 min. After using a piece of filter paper to blot residual sample solution off from the grid edge, samples were observed by Tecnai Spirit 120 kV transmission electron microscope.

### 2.10. Growth Rate Analysis

For monitoring of *Har. hispanica* growth rate, 150 *μ*L of culture normalized to OD_600_ 1.0 was used to inoculate 40 mL of AS-168 medium in a flask and cultured at 37°C with shaking at 200 rpm. AS-168 medium contains 3.4 M NaCl, 2.3 M, and 4.7 M NaCl which are referred to the dosage of sodium chloride (200 g, 135 g, or 275 g NaCl) in 1 L AS-168 medium. Samples were withdrawn at time intervals. Growth was measured spectrophotometrically at an optical density of 600 nm. For each strain, three independent biological repeats were conducted.

### 2.11. Motility Assay

Flagellum-mediated swimming motility was assayed by stab inoculating strains onto AS-168 agar plates (with 0.3% agar) [23]. After 5 days of incubation at 37°C, motility was assessed by measuring the diameters of the circular zones that the colonies spread from their points of inoculation.

### 2.12. Adhesion Assay

For the rapid attachment assays, a saturated culture 150 *μ*L (the stationary growth stage, OD_600 nm_ 2.7) was added to wells of a microtiter dish (Falcon 3911). After incubation at 37°C for 4 days, the planktonic and loosely adherent haloarchaeal cells were washed off, and surface-attached cells were stained by addition of 0.1% crystal violet, solubilized in 95% ethanol, and measured (A_540 nm_) as described previously [24].

## 3. Results and Discussion

### 3.1. An Acidic EPS from *Har. hispanic* Was Isolated and Purified

**Figure 1:**
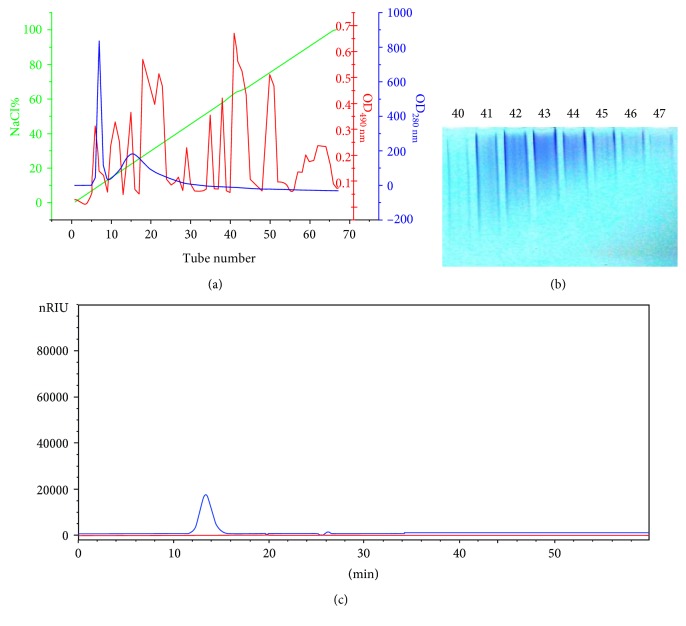
The acidic EPS separation and purification. (a) Separation of the acidic EPS from crude solution by DEAE-Sepharose Fast Flow column. Green curve, NaCl%; blue curve, OD_280 nm_ by UV detector; and red curve, OD_490 nm_ by phenol-sulfuric acid reaction. (b) 7.5% PAGE electrophoresis detection of acidic EPS. (c) Homogeneity analysis of the EPS by HPGPC.

**Figure 2:**
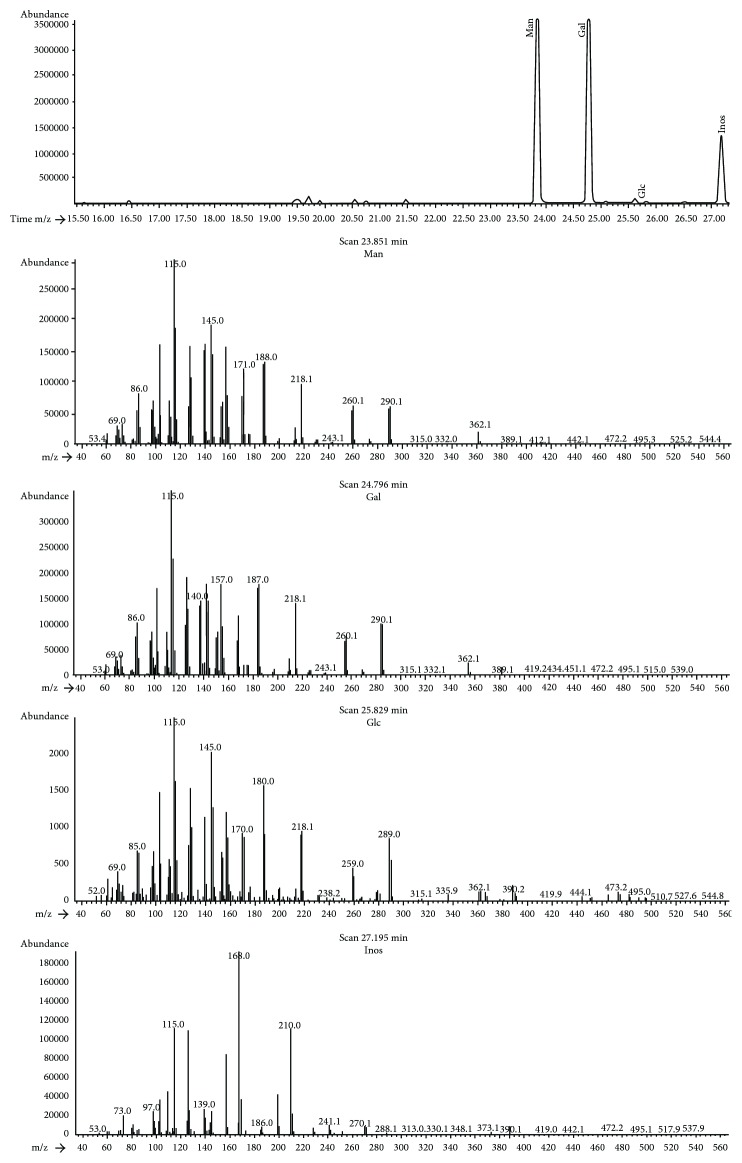
Sugar composition of the acidic EPS from *H. hispanic*. GC-MS results.

The acidic EPS was isolated and purified from AS-168 medium according to Section 2.2; totally 30 mg of the acidic EPS was purified from 1 L of culture medium, and the acidic EPS was purified to homogeneity as judged by HPGPC (Figure 1). The molecular weight was determined as 1100 kDa using dextran markers. GC-MS analysis revealed that the EPS was composed primarily of mannose and galactose with a small amount of glucose with a molar ratio of 55.9 : 43.2 : 0.9 (Figure 2). The total carbohydrate content was determined as 51% (*w*/*w*). We also detected SO_4_^2−^ group using IR spectrum and ion chromatography; the sulfate content was determined as 26% (*w*/*w*). The EPS sample was readily dissolved in water during the composition analysis but did not dissolve well in the DMSO used for the linkage analysis, making the permethylation procedure more difficult. Thus, more work is needed to make clear the linkage structure of the acidic EPS.

The glycosyl composition of the acidic EPS from *Har. hispanic* ATCC 33960 was different from other EPSs reported in halophilic archaea (Table 2). The acidic EPS with high sulfate content might have specific biological functions and give a great potential for application [25], but the amount of EPS produced by *Har. hispanic* ATCC33960 is insufficient to use the biopolymer. If we know the exact synthesis pathway of the biopolymer, especially the critical genes responsible for the EPS synthesis, genetic manipulation can be used to obtain more EPS.

### 3.2. Deletion of *HAH_1662* or *HAH_1667* Leads to Loss of the Acidic EPS

**Figure 3:**
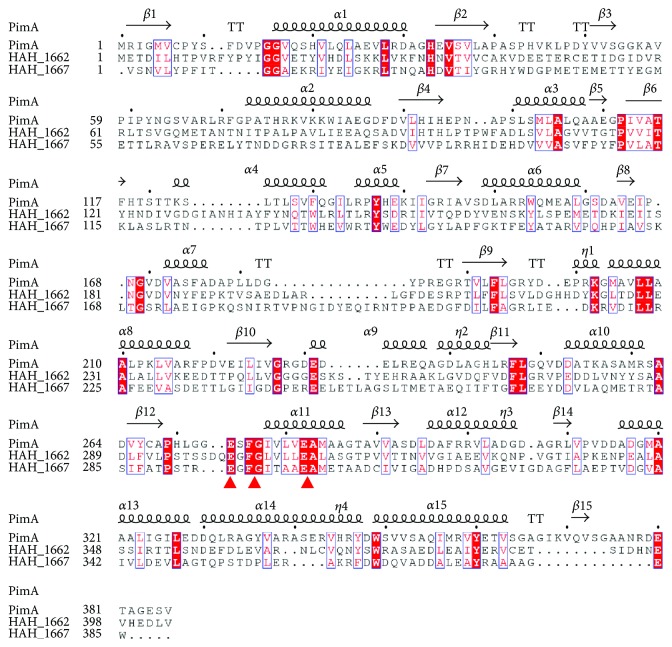
Structural sequence alignment of HAH_1662, HAH_1667, and PimA. The conserved EXF(G/C)X_4_E motifs were labelled with triangles, and the sequences were aligned using Clustal software and ENDscript server.

The genome of *Har. hispanic* ATCC33960 has been completed; HAH_1661, HAH_1662, HAH_1663, and HAH_1667 were all annotated as glycosyltransferases in a polysaccharide biosynthesis gene cluster [26]. HAH_1665, annotated as a polysaccharide biosynthesis protein, and HAH_1666, annotated as an arylsulfatase A family protein, might all together participate in the synthesis of the acidic EPS (Figure S1A available online at https://doi.org/10.1155/2017/5842958). The acidic EPS is rich in mannose, so mannosyltransferases might be critical in the biosynthesis of the acidic EPS. HAH_1662 and HAH_1667 have a highly conserved motif EXF(G/C)X_4_E similar to the mannosyltransferase PimA from mycobacteria [27] (Figure 3()). PimA (PDB accession code 4NC9) is a membrane-associated enzyme that belongs to GT-B superfamily and initiates the biosynthetic pathway of cell wall lipoglycans, using GDP-Man as sugar donor and phosphatidylinositol (PI) as sugar acceptor [28, 29]. So we mainly focused on the two genes *HAH_1662* and *HAH_1667* in this article.

We first detected the expression of *HAH_1662* and *HAH_1667* genes by RT-PCR. As shown in Figure S1B, the *HAH_1662* and *HAH_1667* were actively transcripted during exponential phase growth. To explore its function, the *HAH_1662* and *HAH_1667* were deleted as described in the experimental procedures. The gene deletion mutants, Δ*HAH_1662* and Δ*HAH_1667*, were confirmed by PCR (Figure S1C) and Southern blot (Figure S1D), in which the expected mutant patterns were obtained. In wild-type strain, a 2418 bp fragment containing *HAH_1662* gene and its flanking regions was amplified. In the *HAH_1662* deletion mutant, only a 1224 bp fragment was obtained. In pop-in strain, 2418 bp and 1224 bp fragments were all obtained (Figure S1C left). In wild-type strain, a 2329 bp fragment containing *HAH_1667* gene and its flanking regions was amplified. In the *HAH_1667* deletion mutant, only a 1207 bp fragment was obtained. In pop-in strain, 2329 bp and 1207 bp fragments were all obtained (Figure S1C right). The Southern blot analysis of the Δ*HAH_1662* was carried out by digestion of genomic DNA with EcoR V. The size of hybridization fragments is indicated in the schematic diagram: 3336 bp for wild-type strain and 4375 bp for Δ*HAH_1662* (Figure S1D up). The Southern blot analysis of the Δ*HAH_1667* was carried out by digestion of genomic DNA with EcoR І. The size of hybridization fragments is indicated in the schematic diagram: 4921 bp for wild-type strain and 3799 bp for Δ*HAH_1667* (Figure S1D down).

**Figure 4:**
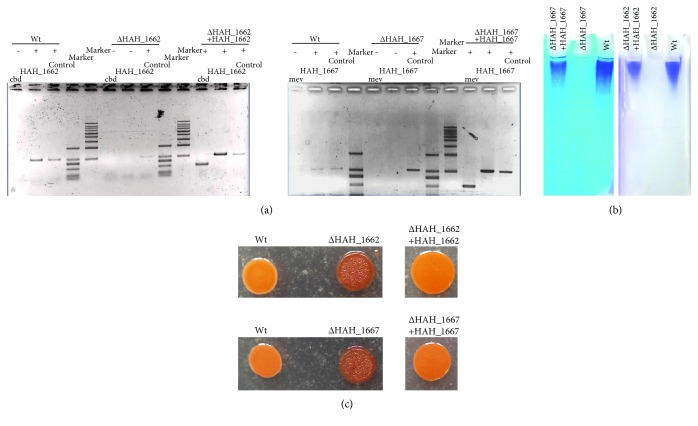
The analysis and verification of the gene complementary strains. (a) RT-PCR analysis of the wild-type, the deletion mutant, and complementary strains. (b) 7.5% PAGE electrophoresis analysis of the acidic EPS extracted from wild-type, gene deletion mutant, and reverted strains. (c) The colony morphology of the wild-type, gene deletion mutant, and complementary strains.

No acidic EPS was extracted from the deletion mutant strains (Figure 4(b)). When the *HAH_1662* and *HAH_1667* genes were reintroduced into the Δ*HAH_1662* and Δ*HAH_1667* mutants as described under experimental procedures, respectively, we verified the transcription of complementary genes by RT-PCR (Figure 4(a)) and the synthesis of the acidic EPS was restored in both complemented strains (Figure 4(b)). The complemented strains cultured on AS-168 plates with antibiotics were able to keep moisture as the wild-type strain (Figure 4(c)), while the mutants were dry and defective in mucoid polymers. These results indicated that both *HAH_1662* and *HAH_1667* genes were responsible for biosynthesis of the acidic EPS in *Har. hispanica*.

### 3.3. The Mutant Strains Displayed Abnormal Cell Surface Morphology

**Figure 5:**
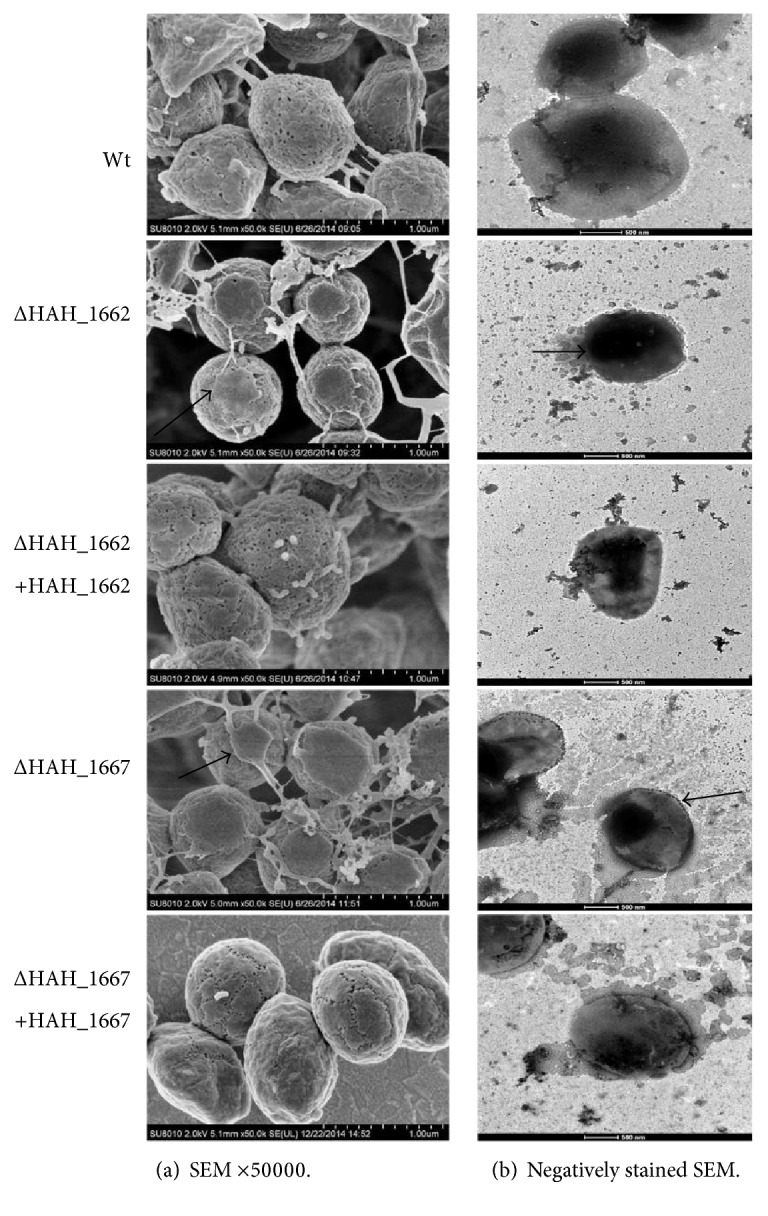
Electron microscopy observation of the wild-type, gene deletion mutant, and gene complementary strains. (a) Scanning electron microscopy analysis of wild-type, gene deletion mutant, and gene complementary strains. ×50000. (b) Negatively stained transmission electron microscopy analysis of wild-type, gene deletion mutant, and gene complementary strains.

Under SEM (Figure 5(a)), both Δ*HAH_1662* and Δ*HAH_1667* mutants displayed a different cell surface morphology (Figure 5(a)) as compared with the wild-type or complemented strains. We can find broken capsules around the mutant cells (the black arrows in Figure 5(a)). In addition to the S-layer, an external capsule was first reported as an outermost cell layer in haloarchaea *Haloquadratum walsbyi* by Sublimi Saponetti et al. [30]. It has been proposed that halomucin, an extremely large protein, might establish the framework of a cross-linked extracellular matrix contributing to the rigidity and maintenance of *H. walsbyi* cell morphology [30–32]. Here, we proposed that the acidic EPS might also serve as a cross-linked extracellular matrix surrounding the S-layer to maintain the cell rigidity and protect the cells against harsh environments. The deficiency of the acidic EPS resulted in an incomplete capsule.

Under the negatively stained TEM (Figure 5(b)), the results were coincident with the SEM results, both Δ*HAH_1662* and Δ*HAH_1667* mutants displayed an abnormal cell surface characteristics. The capsule surrounding Δ*HAH_1662* decreased a lot compared with the wild-type strain but some also existed (the black arrow in Figure 5(b)); the remnant capsule might be other extracellular matrix such as proteins similar to the reported halomucin; the complementary strain of Δ*HAH_1662* almost reverted the cell surface morphology. The capsule surrounding Δ*HAH_1667* became broken and incompact compared with the wild-type strain, so we can see the edge of the Δ*HAH_1667* cell (the black arrow in Figure 5(b)) by negatively stained TEM. Δ*HAH_1662* had a more serious defect in cell surface morphology compared with Δ*HAH_1667*, so we supposed that HAH_1662 might initiate the synthesis of the acidic EPS more like PimA in the mycobacteria [28]. When *HAH_1667* was deleted, some truncated EPS might also be synthesized, acting as an extracellular matrix and forming a broken capsule around Δ*HAH_1667.* The complementary strain of Δ*HAH_1667* did not revert the cell surface morphology well; the reason might be the presence of a complementary plasmid with mevinolin resistance; the mevinolin in AS-168 medium will interfere with lipid synthesis, which might affect the cell membrane and cell wall.

### 3.4. Growth of the Mutants in Different Salty Environments

**Figure 6:**
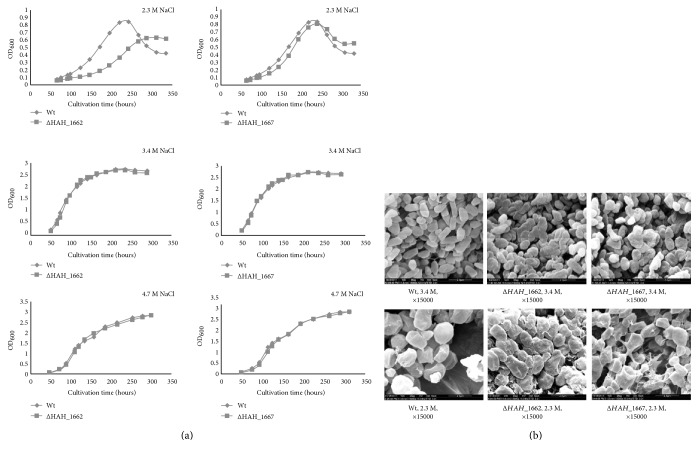
The growth analysis of the wild-type and gene deletion mutant strains. (a) The growth curve of the wild-type and gene deletion mutant strains under different NaCl concentration. (b) Scanning electron microscopy analysis of the wild-type and gene deletion mutant strains under 2.3 M and 3.4 M NaCl concentration.

As shown in Figure 6(a), when cultured in AS-168 medium with low NaCl concentration (2.3 M), Δ*HAH_1662* showed a dramatically retarded growth as compared with the wild-type strain, the growth rate of Δ*HAH_1667* had a little retardation compared with the wild-type strain. The results also indicated that HAH_1662 might play a more important role than HAH_1667 in synthesis of the acidic EPS and initiate the acidic EPS synthesis. When *HAH_1667* was deleted, some truncated EPS might also be synthesized, acting as an extracellular matrix, forming an incomplete capsule, and partially protecting Δ*HAH_1667* against low-salty environment.

When cultured under the most adaptable NaCl concentration (3.4 M) or higher NaCl concentration (4.7 M), the growth rate of the two mutant strains was not affected. The cell morphology of Δ*HAH_1662* and Δ*HAH_1667* was different from the wild-type strain when cultured in 3.4 M AS-168 medium, but the difference was dramatically enhanced when cultured in 2.3 M AS-168 medium (Figure 6(b)), especially Δ*HAH_1662*; they looked more aggregated and swollen, suggesting that the acidic EPS might serve as a protective layer to stabilize the cell surface and maintain the cell morphology.

### 3.5. The Mutants Showed Increased Adhesion and Swimming Ability

**Figure 7:**
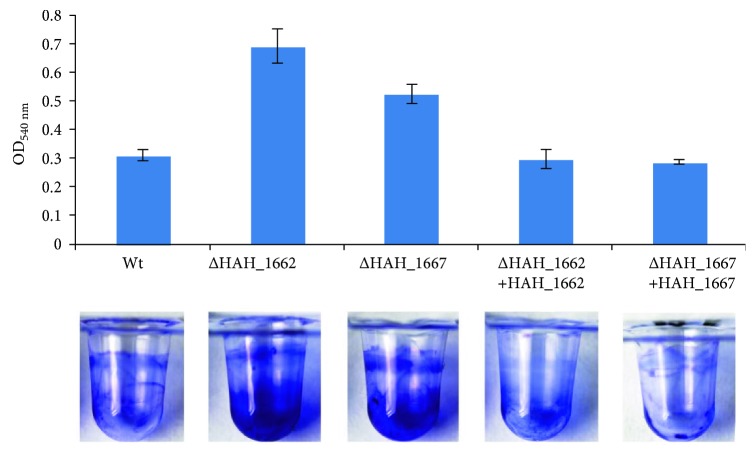
The adhesion analysis of the wild-type, gene deletion mutant, and complementary strains.

**Figure 8:**
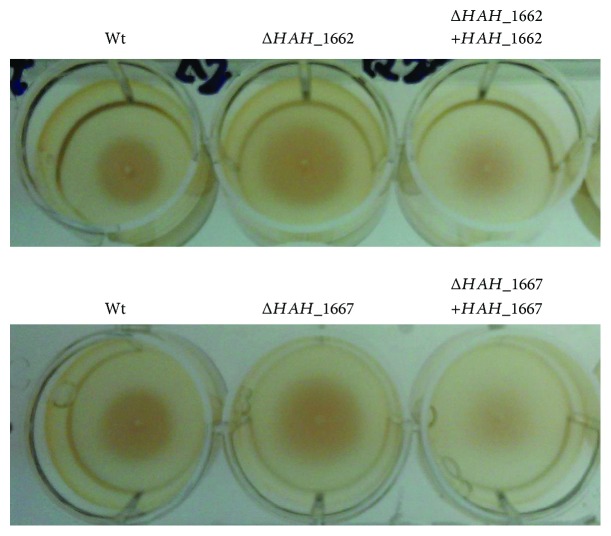
The swimming analysis of the wild-type, gene deletion mutant, and complementary strains.

The two acidic EPS-deficient mutants Δ*HAH_1662* and Δ*HAH_1667* exhibited increased adhesion ability (Figure 7), detected according to experimental procedure in Section 2.12; the mutant strains Δ*HAH_1662* and Δ*HAH_1667* also exhibited increased swimming ability as shown in Figure 8; the swimming zone diameters of the wild-type strains, Δ*HAH_1662* and Δ*HAH_1667*, were, respectively, 2.2 ± 0.1 cm, 3.3 ± 0.1 cm, and 3.2 ± 0.1 cm; the diameters of the complementary strains of Δ*HAH_1662* and Δ*HAH_1667* were, respectively, 2.4 ± 0.1 cm and 2.5 ± 0.1 cm.

EPS biosynthesis and flagella-biosynthesis are usually inversely regulated, so we can understand an increased swimming ability in Δ*HAH_1662* and Δ*HAH_1667*. In bacteria, decreased flagella-dependent motility as well as increased adhesion and EPS production can promote the biofilm formation [24, 33–37]. We found that *Haloarcula hispanic* also could form biofilms when cultured in static chambers, and the gene deletion mutants Δ*HAH_1662* and Δ*HAH_1667* more tended to form biofilms (the air-liquid layer and the bottom layer) as compared with the wild-type strain (supplemental material Figure S2). This was coincident with the increased adhesion ability in the mutant strains. But the mutant strains also displayed an increased swimming ability and a deficiency in EPS synthesis. We know that EPSs are major components in the matrix of biofilm in bacteria and haloarchaea. So it was strange that an increased swimming, the deficiency of EPS, and an increased biofilm formation happened in the mutant strains. One reason may be that we detected the cell motility at the planktonic growth, not at the stage of biofilm formation; the acidic EPS we reported in the article was isolated and detected in the planktonic stage, not at the stage of biofilm formation.

It was reported that some regulations exist among the EPS production, flagella motility, pili adhesion, and biofilm formation in bacteria [35]. But the regulatory networks are not yet known for any biofilm-forming archaeon. Several haloarchaeal species could form a protective nutrient and ion-absorbing mucous biofilms that may help regulate the transport of ions required for the salt-in strategy [8, 38–41]. The two gene deletion mutants Δ*HAH_1662* and Δ*HAH_1667* may be interesting candidates for mechanism research of biofilm formation that is absolutely unclear in haloarchaea.

## 4. Conclusion

In this study, an acidic EPS from *Haloarcula hispanic* ATCC33960 was isolated and purified, which was different from other EPSs reported in haloarchaea. *HAH_1662* and *HAH_1667* were verified to be responsible for the EPS biosynthesis. Deletion of the *HAH_1662* or *HAH_1667* genes led to loss of the acidic EPS and abnormal cell surface morphology. Our results suggest that biosynthesis of the acidic EPS might act as an adaptable mechanism to stabilize the cell surface structure and protect the cells against harsh environments.

## Supplementary Material

The information of supplementary materials are as follows: Fig S1 (A) A putative polysaccharide biosynthesis gene cluster in Har.hispanic ATCC33960. (B) RT-PCR analysis of the expression and deletion of HAH_1662 or HAH_1667 gene. (C) PCR analysis of the pop-in and pop-out strains. (D) Southern blot analysis of the gene deletion mutants. Fig S2 The biofilm formation in a static 12-well plate. (A) The air-liquid biofilms formed by the wild-type, ΔHAH_1662 and ΔHAH_1667 strains. WT(A1,B1,C1); ΔHAH_1662(A3,B3,C3); ΔHAH_1667(A4,B4,C4); Control(A2,B2,C2). (B) The grey column, the bottom biofilms formed by the wild-type, ΔHAH_1662 and ΔHAH_1667 strains; the orange column, the static-cultured strains; OD540nm, the adhesion values of the wild-type, ΔHAH_1662 and ΔHAH_1667 strains.

## Figures and Tables

**Table 1:** Oligonucleotides used in this study.

Primers	Sequences(5′ → 3′)	Restriction site
p1	CCCAAGCTTTATGGCCGAGAACATCCTCG	Hind III
p2	CCGTCGATTGAAACGGTTTGGGAATTAGTAAATTAG	
p3	CTAATTTACTAATTCCCAAACCGTTTCAATCGACGG	
p4	CGGGGTACCCGCATACCTCTTGGTATAG	Kpn І
p5	GGAATTCCATATGGATATCCTCCACACGCC	Nde І
p6	GGGGTACCCTAAACTAAGTCTTCATGTACC	Kpn І
p7	AACCGTTTCAATCGACGGTATATCCTGATTATTC	
p8	ATCTCACAACATCTGTTGATTCTGGCATTCACAAG	
p9	CCCAAGCTTCACTACATCATCCAAACTTC	Hind III
p10	TTCTCTTTTTCGTTGACCAAAAGCGATGTTTGTCATTC	
p11	ATGACAAACATCGCTTTTGGTCAACGAAAAAGAGAACG	
p12	CGGGGTACCTCACGAGACGATCATGG	Kpn І
p13	CGCGGATCCATTTTCCAGGGATCTTTCAAATG	BamH І
p14	GGGGTACCGCTTTGGGGAGATCCGTGTAACTC	Kpn І
p15	GGTCAACGAAAAAGAGAACGTGGCAAGAAGTG	
p16	CCCGGAGGTAATGCACCAGCGATGGCTCGAAAC	
p17	ATGGATATCCTCCACACGCC	
p18	CATGTACCTCGTTATGATCG	
p19	GTGAGTAATGTTCTGTATCC	
p20	GCCCTGTATGCTTCCAGTGC	
p21	ATGGCAAATACACCGGTATCAG	
p22	TACTACACTGCCACCGGGTTC	
p23	ATGACAGACGCCGCGTCCCTC	
p24	CAGGTACACCGAGTTGCCGA	

**Table 2:** A comparison of sugar composition of EPS in haloarchaea.

Haloarchaea	Sugar composition of EPS
*Haloferax mediterranei* ATCC 33500	Man : 2-amino-2-deoxy-GlcA = 1.0 : 1.1 [15]
*Haloferax gibbonsii* ATCC 33959	Man : Glc : Gal : Rha = 2 : 1 : 3 : 1 [10]
*Haloterrigena turkmenica*	Glc : GlcNH_2_ : GlcA : Gal : GalNH_2_ = 1 : 0.65 : 0.24 : 0.22 : 0.02 [11]
*Haloarcula* spp. T5	Man : Gal : GlcA = 2 : 1 : 3 [8]
*Haloarcula* spp. *T6 and T7*	Man : Gal : Glc = 1 : 0.2 : 0.2 [8]
*Haloarcula hispanic* ATCC33960	Man : Gal : Glc = 1 : 0.77 : 0.02
