# Supplementary figures and images for: An Acidic Exopolysaccharide from Haloarcula hispanica ATCC33960 and Two Genes Responsible for Its Synthesis
Source: Archaea. 2017 May 28;2017:5842958. doi: 10.1155/2017/5842958 (PMC5467301; doi:10.1155/2017/5842958)

**
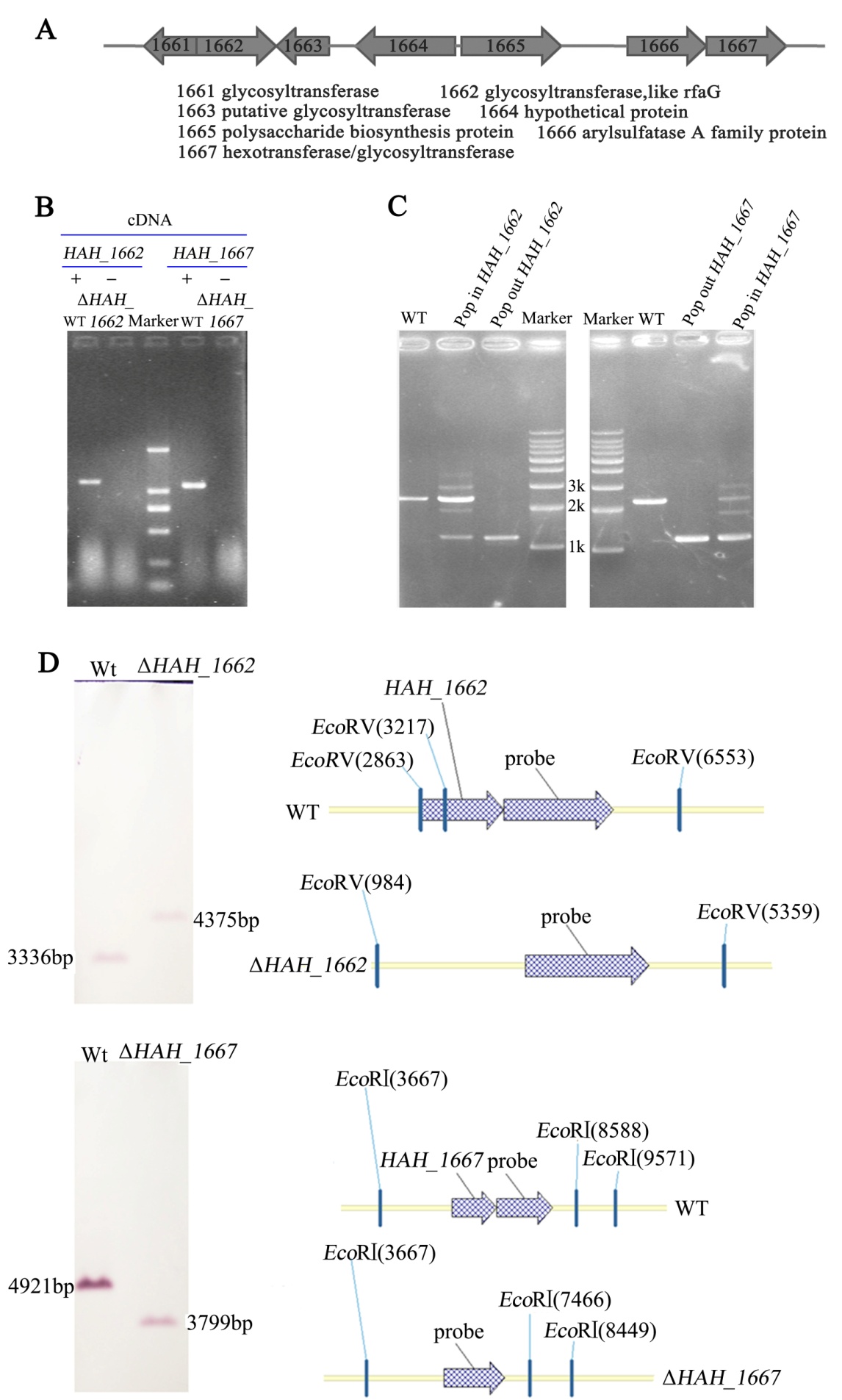
**

Supplement: Supplementary file 1 — The information of supplementary materials are as follows: Fig S1 (A) A putative polysaccharide biosynthesis gene cluster in Har.hispanic ATCC33960. (B) RT-PCR analysis of the expression and deletion of HAH_1662 or HAH_1667 gene. (C) PCR analysis of the pop-in and pop-out strains. (D) Southern blot analysis of the gene deletion mutants. Fig S2 The biofilm formation in a static 12-well plate. (A) The air-liquid biofilms formed by the wild-type, ΔHAH_1662 and ΔHAH_1667 strains. WT(A1,B1,C1); ΔHAH_1662(A3,B3,C3); ΔHAH_1667(A4,B4,C4); Control(A2,B2,C2). (B) The grey column, the bottom biofilms formed by the wild-type, ΔHAH_1662 and ΔHAH_1667 strains; the orange column, the static-cultured strains; OD540nm, the adhesion values of the wild-type, ΔHAH_1662 and ΔHAH_1667 strains. [file 5842958.f1.docx]

A

Δ*HAH_1667*

Δ*HAH_1662*

Wt


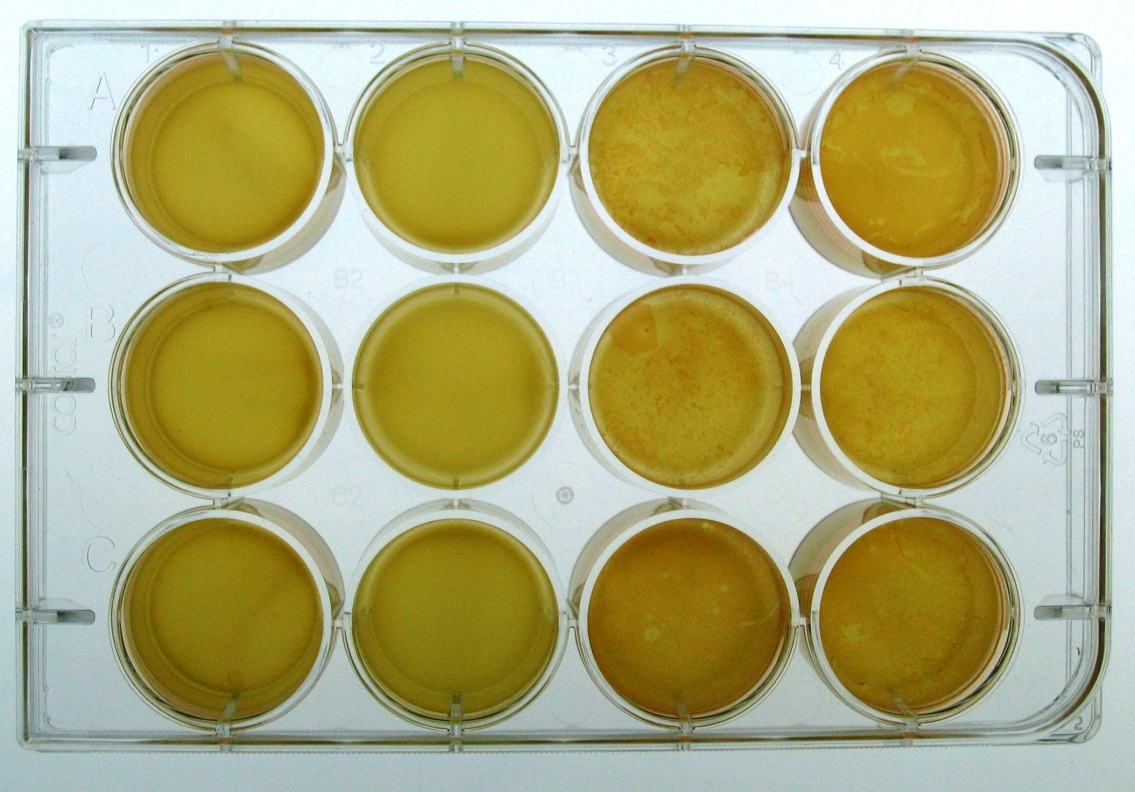


4

3

2

1

C

B

A

B


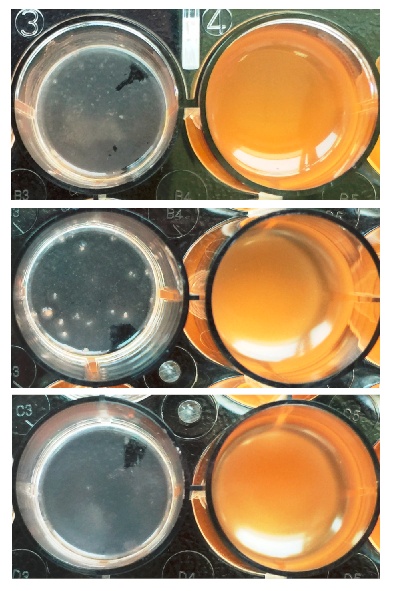


OD540nm 1.04±0.04

OD540nm 1.21±0.08

OD540nm 0.75±0.08

Δ*HAH_1667*

Δ*HAH_1662*

Wt

Supplement: Supplementary file 2 [file 5842958.f2.docx]
